# Supplementary material for: Reproducibility and Feasibility of Classification and National Guidelines for Histological Diagnosis of Canine Mammary Gland Tumours: A Multi-Institutional Ring Study
Source: Vet Sci. 2022 Jul 13;9(7):357. doi: 10.3390/vetsci9070357 (PMC9325225; doi:10.3390/vetsci9070357)
Supplement: Supplementary file 1 [file vetsci-09-00357-s001.zip › vetsci-1733298-supplementary.pdf]

Table S1. Histological grading given by panellists (P) for the 36 canine mammary tumor samples included in the study, majority opinion (GM) for grade is given for those with a majority opinion of malignancy. No GM grade was given to three malignant cases with a subtype GM (see table 5) for which grading is not expected (i.e. squamous cell carcinoma, sarcoma, and carcinosarcoma).

Sample ID	P01	P02	P03	P04	P05	P06	P07	P08	P09	P10	P11	P12	P13	P14	P15	GM grade	GM H/B/M
1			II	II	III	I	I	I	II	II	III		II	I	II	II	M
2	III	II	I	II	I	III	II	II	III	III	II	I	III	I	III	III	M
3			I	I	I								I				B
4	I	II	I	I		I	I	II	II	II	I	I	II	I	II	I	M
5	II	I	II	II	I	II	II	II	II	II	II	II	III	I	II	II	M
6		III	II	II	I	II	II	II	III	III	I	II	II	I	III	II	M
7	I		II		I	I	I	I				I					B
8																	H
9																	H
10	II					I	I		II					I	II		
11	III	III	III	II	I	III	III	II	III	III	III	II	III	II	III	III	M
12					II												H
13			I					I									H
14	II	II	III	III			III	II		II	II	II	II	I		II	M
15								I									B
16		III						II								no grading	M
17		I	III	I		I	II	II		II	II	I	I	I		I	M
18																	H
19																	B
20	III			III										III		no grading	M
21	III	II	III	II		III	III	III		III	II	II	III			III	M
22																	H
23																	H
24	II	II	I	I		II	I	II	III	II	II	I	II	I	III	II	M
25					I											no grading	M
26													II				B
27	II		II	II			II	III		III	II	II		II		II	M
28	II	II	I	II		II	I	II		II	II	II	III	I		II	M
29										I							B
30	I	II	III	I		III	I	II	III	II	I	II	II	I	III	-	M

31	II	I	II	II	I	II	II	III	I	II	I	I	II	I	II	II	M
32			I		I												B
33		I	II	II				I	II	II	I	I	II		II	II	M
34	III		II	II	I	II								I		no grading	M
35																	H
36	III	II	III				II	III			II	III		II		-	M

H, hyperplasia-dysplasia; B, benign tumour; M, malignant tumour; GM, majority opinion
